# Influx of uncommon HIV-1 strains from Eastern Europe and identification of a new unique recombinant strain among young Cypriot MSM in Cyprus

**DOI:** 10.1186/1742-4690-9-S1-P98

**Published:** 2012-05-25

**Authors:** Ioanna Kousiappa, Yiota Lazarou, Katerina M Othonos, Johana Hezka, Leondios G Kostrikis

**Affiliations:** 1 University of Cyprus, Nicosia, Cyprus

## Introduction

The polyphyletic picture of HIV-1 infection in Cyprus is a fact, as new variants and unique recombinant forms were found in the recent past. As part of a growing effort to monitor any changes in the molecular epidemiology of HIV we studied two notable cohorts of the known HIV-1 population diagnosed in 2010 to 2011. Near full-length genome sequencing and phylogenetic analysis was carried out to determine the heterogeneity among HIV-1 strains isolated from patients, 10 originated from eastern European countries (mostly Romania) and 8 young Cypriots (<25 yrs), all men who have sex with men (MSM).

## Materials and methods

Sequence of the near full-length genome was amplified by RT-nested PCR from all HIV-1 seropositives and sequenced. Detailed phylogenetic and bootscanning analyses were performed by MEGA v5.0 to determine phylogenetic associations and subtype assignments. To explore putative recombination patterns in the sequences we performed a bootscanning analysis using Simplot, version 3.5.1.

## Results

Phylogenetic analyses of the obtained viral sequences showed genetic diversity. In the eastern European cohort, subtype F1 was the dominant subtype (40%), followed by subtype C (20%), A1, A2, CRF02_AG, and CRF03_AB (10% each). In the young MSM cohort subtype B was the main subtype (50%), followed by subtype A1 (25%), CRF01_AE (12.5%) and one HIV-1 isolate that was not classified in any known subtype or recombinant form (12.5%). Complete recombination analysis revealed that this isolate had a new recombinant pattern, comprising segments of subtypes A1 and B, and is distinct from any reported recombinant.

## Conclusions

These findings exhibit an influx of infrequent HIV-1 genetic forms from eastern European countries in Cyprus, and a stable circulation of B and A1 subtype among the young Cypriot MSM cohort. A unique recombination event between A1 and B subtypes has occurred and the parental strains seem to be formerly characterized Cypriot MSM patients. For the first time, these data show an impact on the evolutionary progress of HIV-1 epidemic of the island. The significance of this study along with the earlier variable epidemiological status of HIV-1 infection in Cyprus reflects the contribution to HIV classification, and the important implications for HIV-1 disease control and surveillance.

